# High-Density Lipoprotein Function in Exudative Age-Related Macular Degeneration

**DOI:** 10.1371/journal.pone.0154397

**Published:** 2016-05-12

**Authors:** Laura Pertl, Sabine Kern, Martin Weger, Silke Hausberger, Markus Trieb, Vanessa Gasser-Steiner, Anton Haas, Hubert Scharnagl, Akos Heinemann, Gunther Marsche

**Affiliations:** 1 Department of Ophthalmology, Medical University of Graz, Graz, Austria; 2 Institute of Experimental and Clinical Pharmacology, Medical University of Graz, Graz, Austria; 3 Clinical Institute of Medical and Chemical Laboratory Diagnostics, Medical University of Graz, Graz, Austria; University of Cologne, GERMANY

## Abstract

**Purpose:**

High-density lipoproteins (HDL) have long been implicated in the pathogenesis of age-related macular degeneration (AMD). However, conflicting results have been reported with regard to the associations of AMD with HDL-cholesterol levels. The present study is the first to assess HDL composition and metrics of HDL function in patients with exudative AMD and control patients.

**Methods:**

Blood samples were collected from 29 patients with exudative AMD and 26 age-matched control patients. Major HDL associated apolipoproteins were determined in apoB-depleted serum by immunoturbidimetry or ELISA, HDL-associated lipids were quantified enzymatically. To get an integrated measure of HDL quantity and quality, we assessed several metrics of HDL function, including cholesterol efflux capacity, anti-oxidative and anti-inflammatory activities using apoB-depleted serum from study participants.

**Results:**

In our study, we observed that the HDL associated acute phase protein serum amyloid A (SAA) was significantly increased in AMD patients (p<0.01), whereas all other assessed apolipoproteins including ApoA-I, apoA-II, apoC-II, apoC-III and apoE as well as major HDL associated lipids were not altered. HDL efflux capacity, anti-oxidative capacity and arylesterase activity were not different in AMD patients when compared with the control group. The ability of apoB-depleted serum to inhibit monocyte NF-κB expression was significantly improved in AMD patients (mean difference (MD) -5.6, p<0.01). Moreover, lipoprotein-associated phospholipase A2 activity, a marker of vascular inflammation, was decreased in AMD subjects (MD -24.1, p<0.01).

**Conclusions:**

The investigated metrics of HDL composition and HDL function were not associated with exudative AMD in this study, despite an increased content of HDL associated SAA in AMD patients. Unexpectedly, anti-inflammatory activity of apoB-depleted serum was even increased in our study. Our data suggest that the investigated parameters of serum HDL function showed no significant association with exudative AMD. However, we cannot exclude that alterations in locally produced HDL may be part of the AMD pathogenesis.

## Introduction

Cholesterol maintenance in the retina is still poorly understood but needs to be studied to delineate the link between retinal cholesterol and age-related macular degeneration (AMD), an important cause of blindness in the elderly population. High-density lipoproteins (HDL) have long been implicated in the pathogenesis of age-related macular degeneration (AMD) [1]. However, conflicting results have been reported with regard to the associations of AMD with HDL-cholesterol levels [2]. Earlier studies reported that high HDL-cholesterol levels were associated with a reduced risk of AMD [3,4], while in more recent studies no association [2,5–9] or even an inverse association between HDL and AMD was observed [10–12]. Likewise systemic levels of apolipoprotein A1 (apoA-I), the major protein component in HDL, were not associated with AMD [13].

Although there have been reports of HDL-related loci associated with AMD [3–6,14,15], some studies could not confirm these results [2,16,17]. Common genetic variants have been found on the cholesteryl ester transfer protein (CETP) gene, which promotes the transfer of cholesteryl-ester from HDL to very low-density and low-density lipoprotein (LDL), the hepatic lipase gene, which is involved in the metabolism of HDL, the apolipoprotein E (apoE) gene, an apolipoprotein of HDL, and the ATP-binding cassette transporter 1 gene, which mediates the efflux of cholesterol and phospholipids to poorly lipidated HDL [15,18,19].

Therefore, the complexity of HDL composition may indicate that changes in the functionality of HDL rather than serum HDL-cholesterol levels determine its protective activities [20]. Dysfunctional or even pro-inflammatory forms of HDL may, therefore, play a role in the pathogenesis of AMD [20]. In the present study, we sought to assess whether dysfunctional HDL, characterised by a reduced ability to mobilise cholesterol and by impaired anti-oxidative and anti-inflammatory capacities, are linked to the pathogenesis of exudative AMD.

## Patients and Methods

### Patient Recruitment

This study was designed as a case-control study. Blood samples were collected from 29 patients with exudative AMD and 26 control patients after obtaining written informed consent at the Department of Ophthalmology, Medical University of Graz. This study was approved by the institutional review board at the Medical University of Graz and adheres to the tenets of the Declaration of Helsinki. All patients with exudative AMD underwent funduscopic examination and optical coherence tomography. Fluorescein/indocyanine green angiography (Heidelberg Spectralis HRA, Heidelberg Engineering, Heidelberg, Germany) was used to confirm the diagnosis of exudative AMD. All 29 patients previously underwent intravitreal injections of vascular endothelial growth factor (VEGF) inhibitors as a treatment for exudative AMD. Initially all patients received bevacizumab. Three patients were switched to aflibercept, when they showed inadequate response to bevacizumab treatment. As a control group we included age-matched patients without any evidence of AMD presenting at our department for cataract surgery. All control patients underwent funduscopic examination and optical coherence tomography. Control patients showing any signs of AMD (e.g. drusen) were not eligible for study enrolment. Exclusion criteria in exudative AMD and control patients were any evidence of other retinal disorders (such as uveitis, retinal vascular occlusion, diabetic maculopathy or myopic CNV). Patients reporting a history of other chronic inflammatory disorders (such as psoriasis, rheumatoid arthritis, heart attack), history of diabetes type 1 and 2, intake of antihyperlipidemic agents, regular intake of anti-inflammatory drugs, history of severe renal failure, history of severe hepatic dysfunction and a recent infection were excluded. The patients’ medical history was carefully reviewed. All exudative AMD and control patients were measured and weighed and their BMI calculated. Fasting blood samples were collected by antecubital venipuncture into two 9 mL serum tubes (VACUETTE TUBE 9 ml Z Serum Clot Activator). The samples were centrifuged, separated and frozen at -80°C after 30 minutes. Laboratory analyses were performed at the Institute of Experimental and Clinical pharmacology, Medical University Graz.

### ApoB-depletion of serum

ApoB-depleted serum was prepared by addition of 40 μL polyethylene glycol (20% in 200 mmol/L glycine buffer) to 100 μL serum. Samples were incubated at room temperature for 20 minutes and the supernatant recovered after centrifugation (10.000 rpm, 20 minutes, 4°C) as described [21].

### Determination of serum and HDL-lipid composition

Levels of total cholesterol, non-esterified cholesterol, triglycerides, phospholipids, fatty acids (Diasys, Holzheim, Germany) were measured enzymatically. LDL cholesterol was calculated according to the Friedewald equation. HDL-associated lipids were measured in apoB-depleted serum. Cholesterol was measured with enzymatic reagents from WAKO (Neuss, Germany) on a WAKO R30 or Olympus AU640 analyzer.

### Apolipoprotein determination by immunoturbidimetry

ApoA-I, apoA-II, apoB, apoC-II, apoC-III and apoE were determined in apoB-depleted serum by immunoturbidimetry with reagents from Greiner (Flacht, Germany). Analyses were performed on an Olympus AU640 analyzer (Olypmpus Diagnostika, Hamburg, Germany). Serum amyloid A (SAA) was quantified by ELISA (Human SAA, BioSource Europe S.A., Belgium).

### Cholesterol efflux capability of HDL

RAW264.7 macrophages, maintained in DMEM with 10% fetal bovine serum were plated on 48-well plates (300.000 cells/well). Cells were labeled for 24 hours with 1 μCi/ml [^3^H]-cholesterol (Perkin Elmer, Boston, MA, USA). To upregulate ATP-binding cassette transporter A1, cells were stimulated for 6 hours with serum-free DMEM containing 0.3 mmol/L 8-(4-chlorophenylthio)-cyclic AMP (Sigma, Darmstadt, Germany). After labeling, cells were washed and [^3^H]-cholesterol efflux was determined by incubating cells for 4 hours with 2.8% apoB-depleted serum. Cholesterol efflux was expressed as the radioactivity in the medium relative to total radioactivity in medium and cells. All steps were performed in the presence of 2 μg/ml of the acyl coenzyme A cholesterol acyltransferase inhibitor Sandoz 58–035 (Sigma, Darmstadt, Germany).

### Dihydrorhodamine (DHR) oxidation

ApoB-depleted serum (1 μL) was placed in a 384-well, 15μl of 50 μmol/L DHR reagent containing 1 mmol/L 2,2'-azobis-2-methyl-propanimidamide-dihydrochloride was added. The increase in fluorescence (538nm) per minute was determined for samples containing only DHR and for samples containing DHR and individual HDL samples.

### Arylesterase activity

Ca^2+^-dependent paraoxonase activity was determined with a photometric assay using phenylacetate as substrates. ApoB-depleted serum was added to 200 μL buffer containing 100 mmol/L Tris, 2 mmol/L CaCl_2_ (pH 8.0) and 1 mmol/L phenylacetate. The rate of hydrolysis of phenylacetate was monitored by the increase of absorbance at 270 nm and readings were taken every 15 seconds at room temperature to generate a kinetic plot. The slope from the kinetic chart was used to determine ΔAb_270nm_ / min. Enzymatic activity was calculated with the Beer-Lambert Law from the molar extinction coefficient of 1310 L*mol^-1^*cm^-1^.

### Ability of serum HDL to inhibit monocyte NF-κB expression

U937 monocytic cells containing a 5x NF-κB- green fluorescence protein reporter cassette were cultivated in RPMI 1640 containing 7.5% fetal bovine serum in 1.1 mL micro tubes (Bioquote, York, UK) (50.000 cells/tube). The cells were pretreated for 1 ½ hours with 2.5% full serum, 5% apoB-depleted serum, 10% LPDS or rHDL (50 μg/ml). Subsequently, the cells were stimulated for 24 hours with lipopolysaccharide (LPS) (50 ng/ml) (Sigma, Darmstadt, Germany), collected by centrifugation at 400 × g for 7 minutes and fixed with 100 μL BD CellFIX solution (BD Biosciences, Franklin Lakes, NJ, USA). The expression of NF-κB was assessed by flow cytometry. The supernatants were collected and used for cytokine quantification by flow cytometry using a multiplex bead-based immunoassay (eBioscience, San Diego, CA, USA).

### Lipoprotein-associated phospholipase A2 (Lp-PLA2) activity

Lp-PLA2 activity in apoB-depleted serum was measured with a commercially available photometric assay (Cayman Europe, Talinn, Estonia) using 2-thio PAF as substrate as described [22].

### Statistics

Our study sample size (n = 29 vs n = 26) was designed to provide ≥ 90% power to detect a 10% difference in cholesterol efflux capability of HDL based on our hypothesis that we would observe differences similar to those described in our previous study.[22] All statistical analyses were performed using STATA 13 (StataCorp LP, USA). Our specific aims were HDL efflux capacity, DHR oxidation, NF-κB, AAE-acitivity and Lp-PLA_2_ activity. We compared the means between the groups using Mann Whitney U and Wilcoxon signed rank test as required. P-values of 0.05 were considered to be significant throughout the manuscript.

## Results

### Demographic Results

Demographics of AMD patients and controls are shown in Table 1. The mean age of the study group was 76 years. There were more females than males participating in this study. The median values of the laboratory parameters were all in the normal range, except for total cholesterol, and not significantly different between the AMD patients and controls. 12 AMD patients were under current antioxidant micronutrients supplementation. The average HDL-cholesterol levels were comparable between exudative AMD patients and controls (50.7 mg/dl vs 52 mg/dl, respectively; mean difference (MD) 1.3, 95% CI -9.6 to 10.9, p = 0.78). After adjusting for age, gender, BMI, CRP level, kidney and liver parameters, and HbA1c, these differences remained statistically not significant (p = 0.6) (Table 2).

**Table 1:** Clinical characteristics of study subjects. Values are reported as median (±SD).

	AMD patients (n = 29)	Controls (n = 26)	*p-value*	*Normal range*
Age (years)	75.6 (±5.8)	77.9 (±5.8)	0.14	*n/a*
Female (Male)	24 (5)	16 (10)	0.08	*n/a*
BMI	26.6 (±4.3)	26.5 (±2.6)	0.88	*n/a*
Creatinine (mg/dl)	0.88 (±0.17)	0.88 (±0.19)	0.98	*<1*
GFR (ml/min)	69.2 (±14.3)	70.7 (±14.4)	0.69	*80–140*
GGT (U/l)	30.3 (±16.8)	24.5 (±13.1)	0.17	*<38*
AST (U/l)	23.7 (±4.4)	23.9 (±6.7)	0.84	*<30*
ALT (U/l)	20.2 (±6.2)	17.2 (±6.9)	0.1	*<30*
HbA1c (mmol/mol)	38.2 (±3.5)	37.4 (±3.3)	0.38	*20–40*
CRP (mg/l)	4.5 (±3.2)	2.4 (±1.4)	0.06	*<5*
Cholesterol (mg/dl)	211 (±34)	204 (±35)	0.55	*<200*
LDL (mg/dl)	124 (±31)	119 (±43)	0.6	*<160*
Triglycerides (mg/dl)	110 (±51)	107 (±44)	0.83	*<150*
C3 (g/l)	1.14 (±0.14)	1.13 (±0.18)	0.83	*0*.*9–1*.*8*
C4 (g/l)	0.27 (±0.06)	0.24 (±0.06)	0.08	*0*.*1–0*.*4*

Values are reported as median (±SD).

**Table 2:** HDL serum levels, efflux capacity and HDL associated enzyme activities.

	AMD patients	Controls	*p-value*	adjusted p-value^a^
HDL-cholesterol (mg/dl)	50.7 (±2.9)	52 (±3.4)	0.78	0.6
HDL efflux capacity (%)	14.1 (±2.4)	13.8 (±1.7)	0.58	0.51
DHR (%)	41.2 (±6.4)	42.9 (±5.6)	0.08	0.39
AE activity (mM/min/ml)	243 (±60)	215 (±63)	0.84	0.5
Inhibition of NF-κB (%)	32.8 (±5.1)	27.2 (±8.6)	<0.05	<0.05
Lp-PLA_2_ activity (mM/min/ml)	169 (±25)	199 (±28)	<0.01	<0.01
ApoA-I (mg/dl)	133 (±5.2)	131 (±4.8)	0.74	0.85
SAA (mg/dl)	8.5 (±1.5)	3.2 (±0.46)	<0.01	<0.01

Values are reported as mean (±SD).

^a^Adjusted for age, gender, BMI, CRP level, kidney and liver parameters and HbA1c.

### HDL-composition

**Fig 1:**
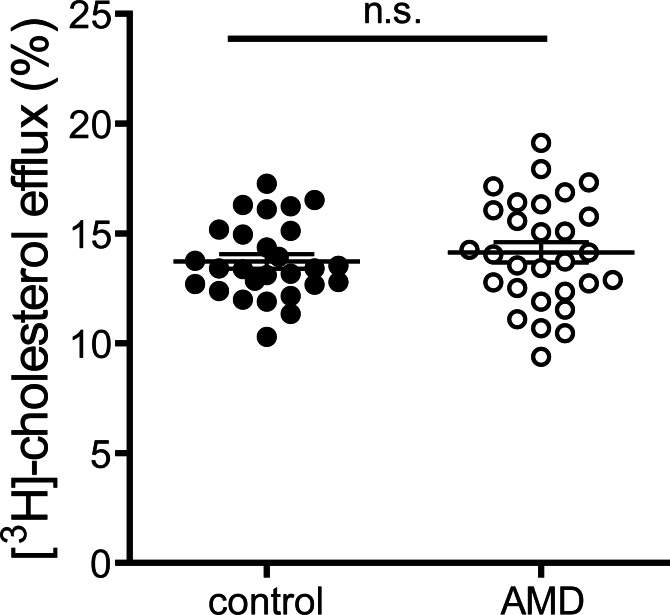
HDL-apolipoproteins and HDL associated lipids. Levels of total cholesterol (A), non-esterified cholesterol (FC) (B), phospholipids (PL) (C), free fatty acids (FFA) (D), triglycerides (TG) (E) were measured enzymatically in apoB depleted serum. HDL associated apolipoproteins ApoA-I (F), apoA-II (G), apoC-II (H), apoC-III (I) and apoE (J) were determined in apoB-depleted serum by immunoturbidimetry.

**Fig 2:**
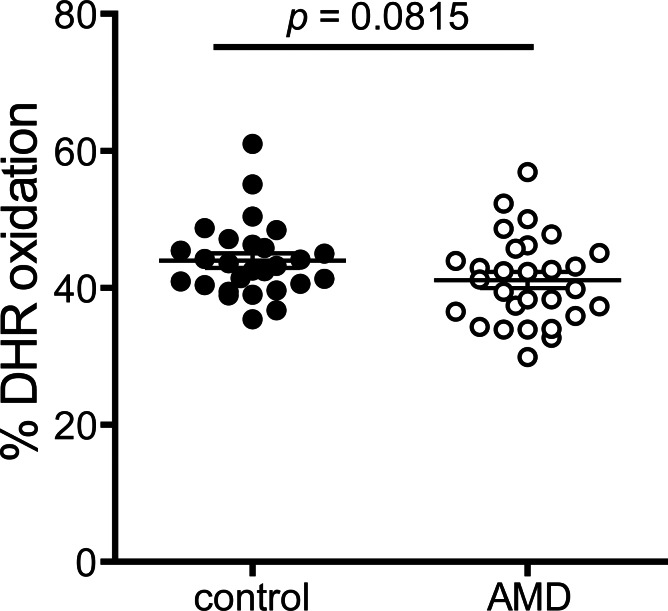
Serum amyloid a levels are increased in AMD patients. Serum amyloid A (SAA) levels in apolipoprotein B (apoB)-depleted sera was quantified by ELISA. Values shown represent means of four independent experiments.

It is now well accepted that different disease states markedly alter HDL composition and function [23]. We assessed major HDL associated lipids and apolipoproteins in apoB-depleted sera of study participants, including apolipoprotein (apo)A-I, apoA-II, apoC-II, apoC-III, apoE and the acute phase protein serum amyloid A(SAA) (Fig 1, Fig 2). As expected, the most abundant proteins on HDL particles from healthy controls and AMD patients were apoA-I and apoA-II (Fig 1). Low-density lipoprotein associated apoB could not be detected, indicating efficient apoB-depletion. After statistical analysis, we identified that the content of HDL associated SAA was significantly increased (Fig 2, p<0.01). After adjusting for age, gender, BMI, CRP level, kidney and liver parameters, and HbA1c, these difference remained statistically significant (p<0.01) (Table 2). All other assessed HDL-apolipoproteins and HDL-associated lipids were not altered in AMD patients (Fig 1).

### Cholesterol efflux capacity

**Fig 3:**
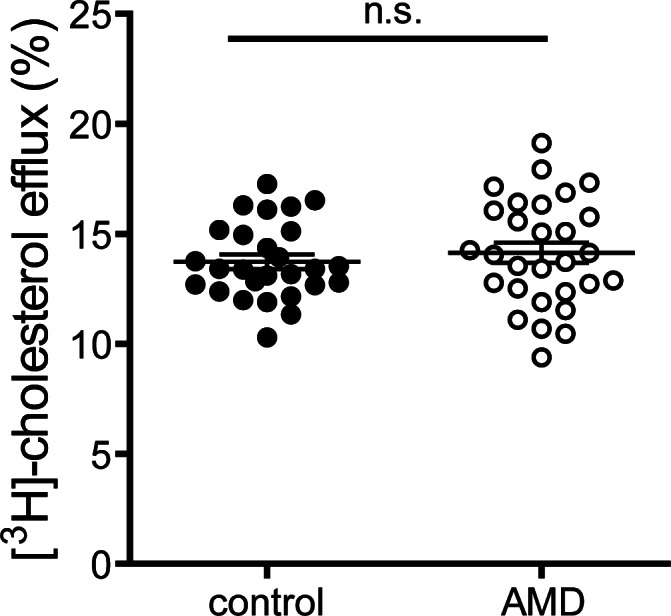
Cholesterol efflux capacity. Apolipoprotein B (apoB)-depleted sera of healthy subjects (control, n = 27) and patients with age-related macular degeneration (AMD, n = 29) were examined for (A) their ability to promote [^3^H]-cholesterol efflux from macrophages. [^3^H]-cholesterol-labeled RAW264.7 macrophages were incubated with 2.8% apoB-depleted sera for 4 hours. Cholesterol efflux is expressed as radioactivity in the supernatant relative to total radioactivity (in supernatant and cells). Values shown represent means of two independent experiments.

Cholesterol efflux capacity of apoB-depleted serum is an integrated measure of HDL quantity and quality [21]. Interestingly, despite alterations in HDL associated SAA (Fig 3), cholesterol efflux capacity of apoB-depleted sera of exudative AMD patients was not altered when compared to controls (MD 0.32, 95% CI -0.8 to 1.5, p = 0.58) (Fig 1). These results prevailed even after adjusting for age, gender, BMI, CRP level, kidney and liver parameters and HbA1c (p = 0.51). Serum efflux capacity was inversely associated with CRP levels (p<0.01) and HDL-associated SAA (p<0.01), in line with previous studies showing that inflammation alters HDL efflux capacity.[24] Higher HDL contents of apoA-I, apoA-II, cholesterol and phospholipids were strongly associated with an increase in efflux capacity (p<0.01).

### Metrics of anti-oxidative activities

**Fig 4:**
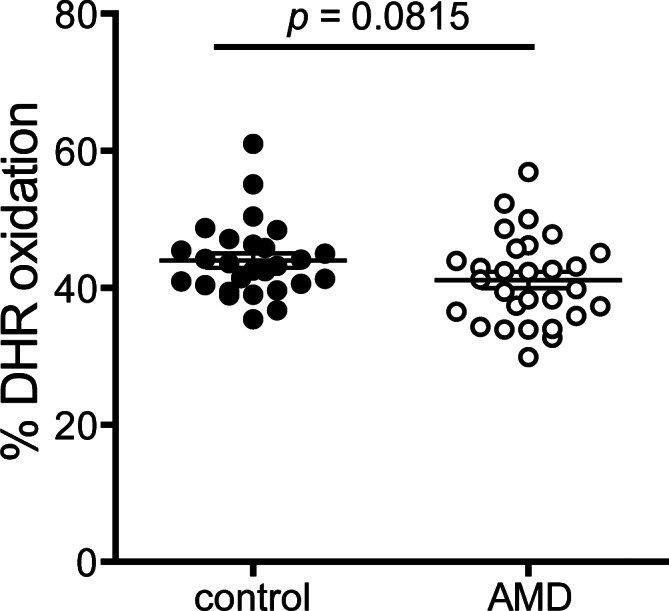
Anti-oxidative capability. The anti-oxidative activity of HDL was determined by inhibition of AAPH-initiated oxidation of the fluorescent dye dihydrorhodamine (DHR). Incubation of DHR in presence of apoB-depleted sera from healthy subjects or AMD patients led to a reduction in the oxidation of DHR. Values shown represent means of two independent experiments.

Anti-oxidative activity of apoB-depleted serum of AMD patients was not significantly different when compared to controls (Fig 4). The average DHR-oxidation rate was 41.2% in AMD patients and 42.9% in the control group (MD -2.7, 95% CI -6.0 to 0.5, p = 0.08). After adjustment for age, gender, BMI, CRP level, kidney and liver parameters and HbA1c, the difference between the groups remained insignificant (p = 0.39). In line with our previous results [25], age was significantly inversely associated with anti-oxidative activity (p<0.01).

**Fig 5:**
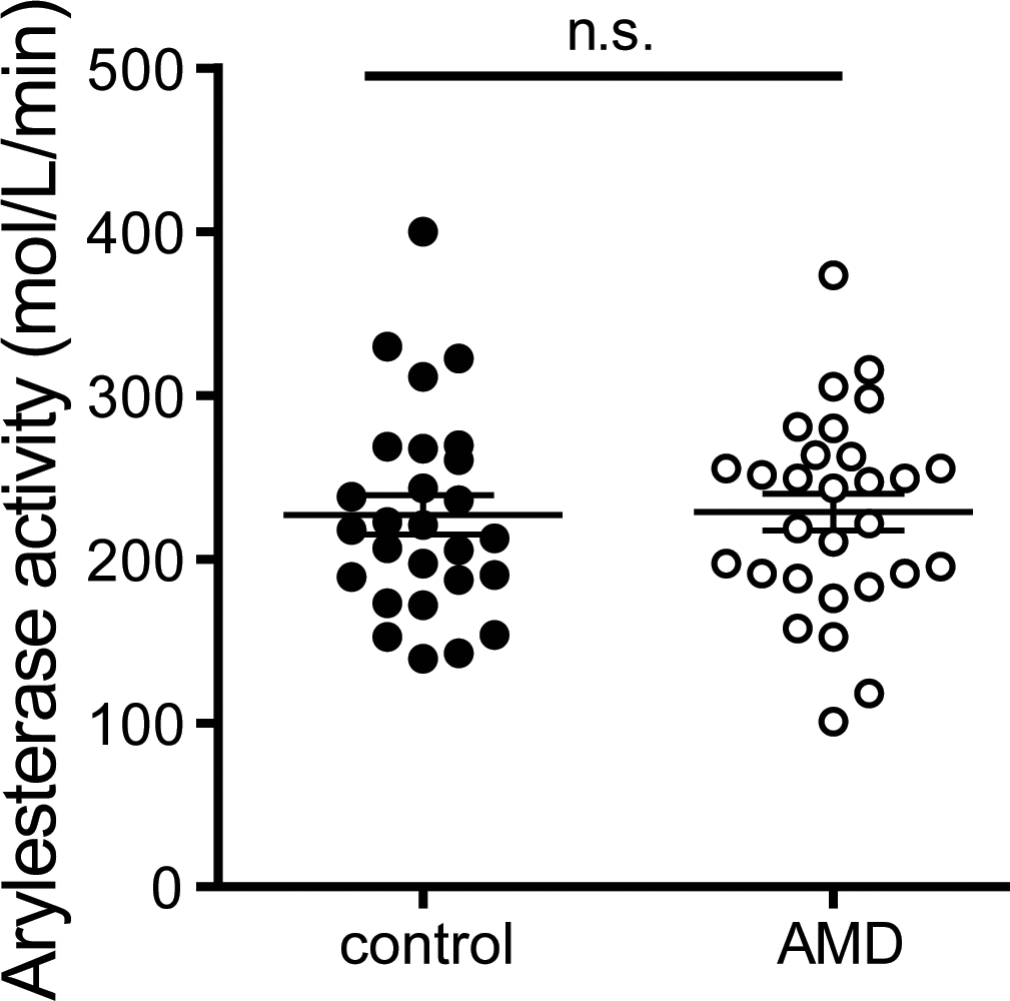
Paraoxonase activity. Activity of HDL-associated paraoxonase was measured using phenylacetate as substrate. Paraoxonase activity of apoB-depleted sera was calculated from the slopes of the kinetic chart. Values shown represent means of four independent experiments.

The average arylesterase activity of PON1 was 242.6 mM/min/ml in AMD patients compared to 215.4 mM/min/ml among controls (MD 3.3, 95% CI -29.9 to 36.7, p = 0.84) (Fig 5). These results prevailed after adjusting for age, gender, BMI, CRP level, kidney and liver parameters and HbA1c (p = 0.5). Higher HDL contents of apoA-I, apoC-III, and triglycerides were associated with an increase in arylesterase activity (p<0.05).

### Metrics of anti-inflammatory activities

**Fig 6:**
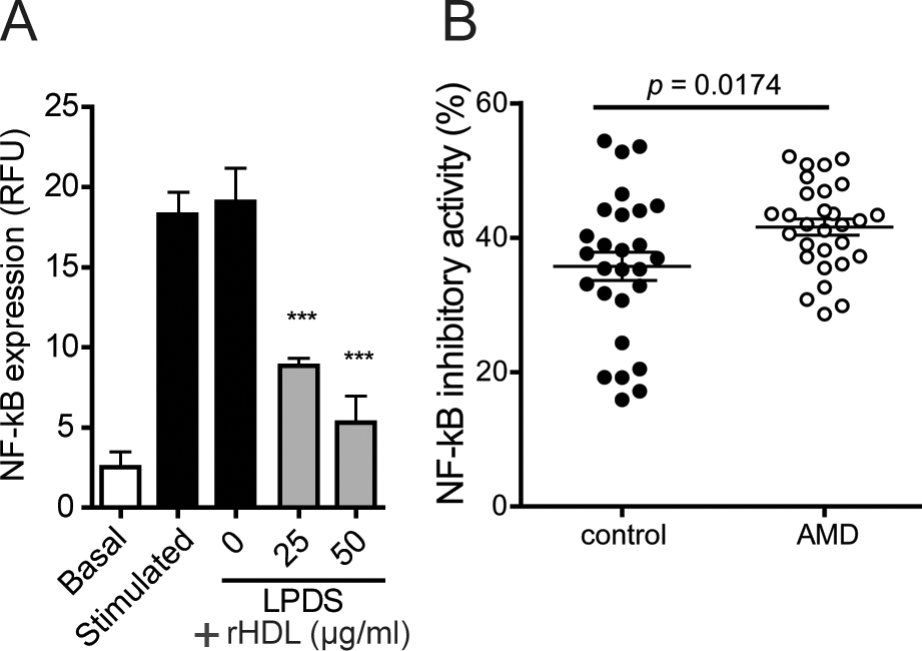
Anti-inflammatory capacity. (A) U937 monocytes containing a reporter cassette for factor-κB (NF-κB) were pretreated in the absence and presence of 10% lipoprotein deficient sera (LPDS) in the presence of indicated concentrations of reconstituted HDL (rHDL). After 1 ½ hours, cells were stimulated with LPS (50 ng/ml) for 24 hours, followed by assessment of GFP expression by flow cytometry. (B) ApoB-depleted sera of healthy subjects and AMD patients were analyzed for their ability to inhibit lipopolysaccharide LPS-induced NF-κB activation in monocytes. U937 monocytes were pretreated with 7% apoB- depleted sera. After 1 ½ hours, cells were stimulated with LPS (50 ng/ml) for 24 hours, followed by assessment of GFP expression by means of flow cytometry. Values shown represent means of two independent experiments.

We assessed the capacity of apoB-depleted sera to inhibit LPS induced activation of the pro-inflammatory transcription factor NF-κB in monocytes. Addition of reconstituted HDL (containing human apoA-I and phosphatidylcholine as sole constituents) effectively and dose-dependently inhibited LPS induced NF-κB activation (Fig 6A) whereas lipoprotein deficient serum (LPDS) showed no inhibitory activity. These data clearly suggest that HDL is required to suppress LPS-induced activation of NF-κB in monocytes. The ability of HDL to inhibit NF-κB expression was higher in AMD patients (MD -5.6, 95% CI -9.5 to -1.8, p<0.05) (Fig 6B), even after adjusting for age, gender, BMI, CRP level, kidney and liver parameters, and HbA1c (p<0.05). NF-κB inhibitory activity was associated with HDL-cholesterol levels (p = 0.04) and tended to be associated with HDL efflux capacity (p = 0.08).

**Fig 7:**
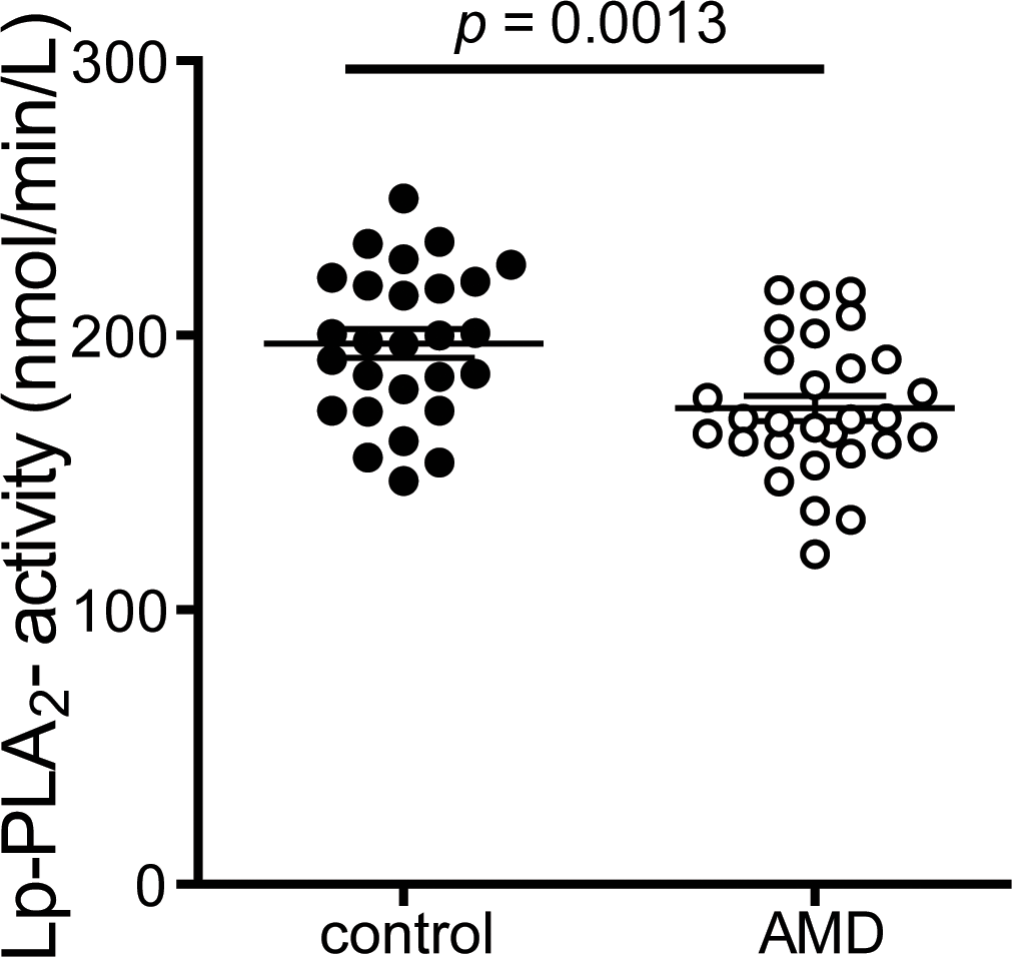
Lipoprotein associated phospholipase A2 (Lp-PLA2) activity. Lipoprotein associated phospholipase A2 (Lp-PLA2) activity of apoB-depleted sera was measured using 2-thio PAF as substrate. Lp-PLA2 activity of apoB-depleted sera was calculated from the slopes of the kinetic chart. Values shown represent means of two independent experiments.

The average Lp-PLA_2_ activity was lower in AMD patients (169.4 mM/min/ml) when compared to controls (198.9 mM/min/ml) (Fig 7). This difference was significant (MD -24.1, 95% CI -38.3 to -9.8, p<0.01), even after adjustment for age, gender, BMI, CRP level, kidney and liver parameters, and HbA1c (p<0.01). Lp-PLA_2_ activity inversely correlated with the ability to inhibit monocyte NF-κB expression (p<0.01).

### Supplemental antioxidant micronutrients

The median NF-κB inhibitory activity was higher in patients currently taking supplemental antioxidant micronutrients, although this was not significant (MD 3.6, 95% CI -1.6 to 8.7, p = 0.17). Current intake of supplemental antioxidant micronutrients was associated with a lower Lp-PLA_2_ activity (MD -25.3, 95% CI -7.9 to -42.7, p<0.01). Age, gender, BMI, CRP levels, kidney and liver parameters, HbA1c, HDL serum levels, HDL efflux capacity, DHR, AE activity and apoA-I levels were not associated with supplemental antioxidant micronutrients intake.

## Discussion

In the present study, we sought to assess whether dysfunctional HDL, characterized by a reduced ability to mobilize cholesterol and by impaired anti-oxidative and anti-inflammatory capacities, are linked to the pathogenesis of exudative AMD. To get an integrated measure of HDL quantity and quality, we assessed several metrics of HDL function, including cholesterol efflux capacity, anti-oxidative and anti-inflammatory activities using apoB-depleted serum from study participants. We assessed major HDL associated lipids and apolipoproteins, including apoA-I, apoA-II, apoC-II, apoC-III, apoE and the major acute phase protein SAA.

We observed that the content of HDL associated SAA was significantly increased in AMD patients, whereas the content of all other assessed apolipoproteins and lipids were not altered. During an inflammatory response, SAA can displace apoA-I from the HDL surface and in extreme circumstances SAA can account for up to 80% of the HDL proteins [26,27]. The contribution of HDL-bound SAA to HDL dysfunctionality is a matter of debate. Previous studies indicated that the ability of HDL to remove cholesterol from human macrophages is only significantly attenuated, when SAA constitutes at least 50% of total protein in HDL [28], a situation that is not achieved in patients with exudative AMD, at least in our study. However, we observed a negative correlation of SAA levels and cholesterol efflux capacity (p<0.01), suggesting that SAA affects cholesterol efflux capacity at least to some extent. SAA was not associated with anti-oxidative or anti-inflammatory activities of apoB-depleted sera.

Although reverse cholesterol transport from macrophages represents only a small fraction of overall cholesterol efflux, it is probably the most relevant factor in atheroprotection [22,29]. The results of our study do not provide evidence of impaired serum cholesterol efflux capacity in exudative AMD patients.

Besides the central role in lipid metabolism, HDL exhibits unique anti-oxidative activity. We assessed the anti-oxidative activity of HDL by measuring increasing fluorescence due to DHR oxidation over time [22]. We found no difference in DHR oxidation between exudative AMD patients and the control group.

As a second marker for anti-oxidative activity of HDL, arylesterase activity of apoB depleted sera, as a marker of HDL associated PON-1 activity, was assessed. In our study, we did not observe that patients with exudative AMD show decreased arylesterase activity of apoB depleted sera. Polymorphisms in the PON-1 gene are associated with AMD [30–32].

HDL is a potent endogenous inhibitor of inflammatory responses [33]. We assessed the capacity of our patients’ sera to inhibit lipopolysaccharide (LPS)-induced activation of the pro-inflammatory transcription factor NF-κB in monocytes. In the absence of added reconstituted HDL, lipoprotein deficient serum shows no inhibitory activity. Addition of reconstituted HDL to lipoprotein deficient serum effectively and dose-dependently inhibits LPS induced NF-κB activation (Fig 5A). ApoB-depleted sera of exudative AMD patients had an increased ability to supress LPS induced NF-κB expression in monocytes when compared to the control group.

We assessed the lipoprotein associated phospholipase A2 (Lp-PLA_2_), a proatherogenic enzyme, as a second marker for HDL-associated anti-inflammatory activity [34]. HDL associated Lp-PLA_2_ activity was significantly lower in exudative AMD patients when compared to control patients.

In our study, patients with AMD showed a significantly greater HDL-associated anti-inflammatory activity when compared with control subjects. These unexpected findings might be explained by the use of antioxidant micronutrient supplementation (containing lutein/zeaxanthin) in exudative AMD patients. Indeed, current intake of supplemental antioxidant micronutrients was associated with a lower Lp-PLA_2_ activity (p<0.01) and the median NF-κB inhibitory activity was higher in patients currently taking supplemental antioxidant micronutrients, although this was not significant. Age, gender, BMI, CRP levels, kidney and liver parameters, HbA1c, HDL serum levels, HDL efflux capacity, DHR, AE activity and ApoA1 were not associated with supplemental antioxidant micronutrients intake.

The repeated use of vascular endothelial growth factor (VEGF) inhibitors [35] might additionally contribute to the greater HDL associated anti-inflammatory activity in exudative AMD patients. Future prospective studies are warranted to elucidate whether supplemental anti-oxidants are able to improve HDL composition and functionality.

We acknowledge limitations to this study. Due to the laborious analyses we kept the patient number rather small. Our study (n = 29 vs n = 26) provided ≥ 90% power to detect a 10% difference in cholesterol efflux capability of HDL based on our hypothesis that we would observe differences similar to those described in previous studies [22]. Much larger studies are warranted to confirm our findings. Although we assessed multiple metrics of HDL function, it is possible that other activities mediated by HDL, like endothelial protective activities, are affected in AMD patients.

Further we did not take genetic polymorphism into account. Genome-wide association studies have identified 21 common genetic variants for age-related macular degeneration, some of which are implicated in HDL function. As we did not perform genetic analysis, certain common and rare genetic variants may influence HDL function and contribute to the development of AMD [15]. In a recent study [14] CETP and apoE genotype influenced HDL cholesterol and apoA-I levels and both were significantly associated with AMD. Therefore future studies are warranted to determine whether genetic polymorphisms affect metrics of HDL functionality.

The retina maintains cholesterol homeostasis by balancing cholesterol input and output. The relative contribution of these pathways to the retinal cholesterol pool is unknown. We cannot exclude that proteins involved in lipoprotein metabolism as well as HDL like particles that are expressed locally within the eye are affected in AMD patients. This might be of particular relevance, given that intra-ocular gene expression suggests production of apoB-containing lipoproteins by the retinal pigment epithelium [13,36,37]. Moreover, we only included patients with exudative AMD. Jonasson et al [20] showed in a study that plasma HDL-cholesterol was associated with incident early AMD and progression to geographic atrophy, but not with exudative AMD.

To our knowledge, this is the first study that assessed parameters of HDL composition and function in AMD patients. The investigated parameters of HDL function were not associated with exudative AMD, despite an increased content of HDL associated SAA in AMD patients. Unexpectedly, anti-inflammatory activity of apoB-depleted serum was even increased in our study. It appears that this is a result of the treatment of patients with micronutrient supplementation rather than disease mediated. In conclusion, the investigated parameters of serum HDL function showed no significant association with exudative AMD. [15]
