# Correction: High-Density Lipoprotein Function in Exudative Age-Related Macular Degeneration

**DOI:** 10.1371/journal.pone.0157210

**Published:** 2016-06-03

**Authors:** Laura Pertl, Sabine Kern, Martin Weger, Silke Hausberger, Markus Trieb, Vanessa Gasser-Steiner, Anton Haas, Hubert Scharnagl, Akos Heinemann, Gunther Marsche

Figs 1 and 2 are incorrectly duplicated from Figs 3–4. The authors have provided corrected versions here.

**Fig 1:**
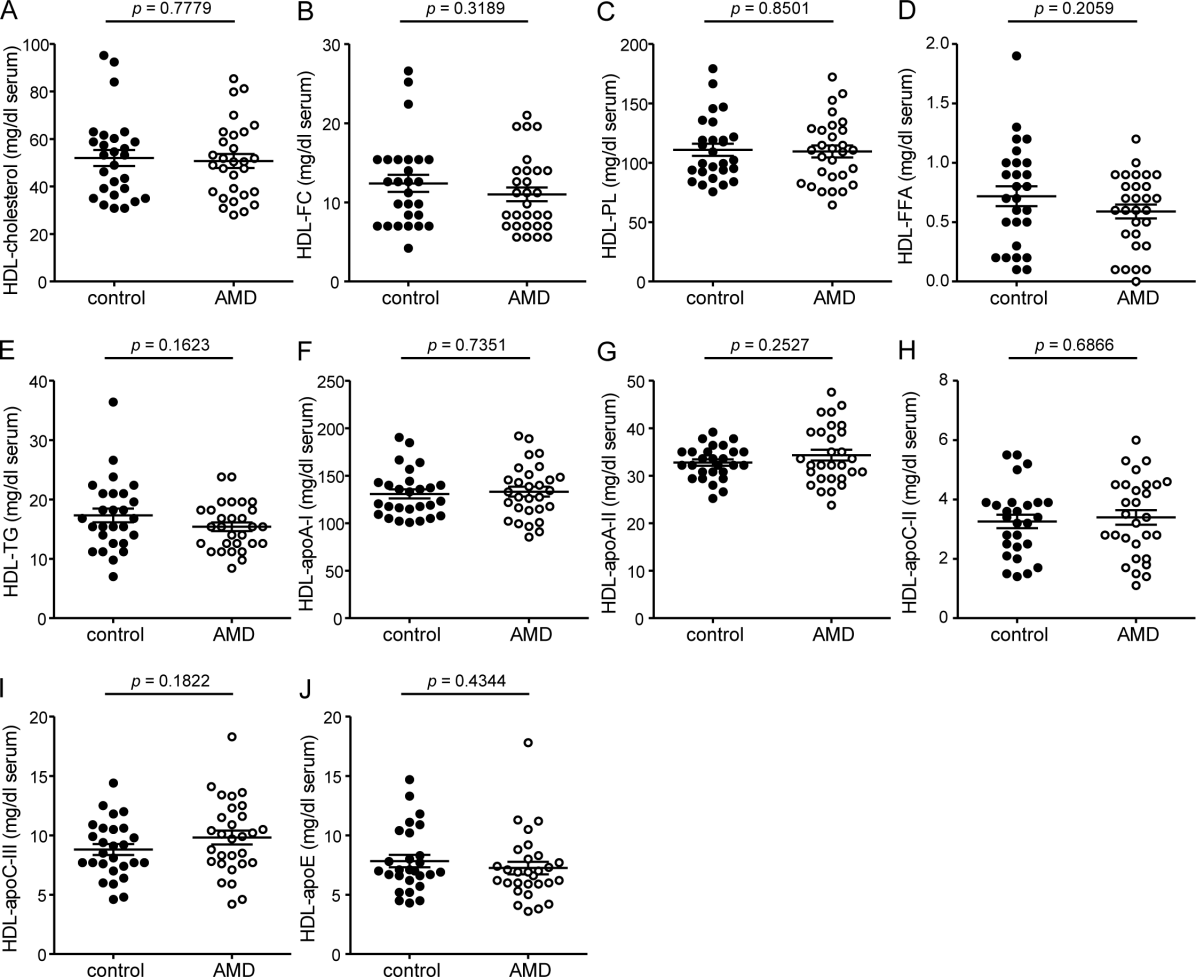
HDL-apolipoproteins and HDL associated lipids. Levels of total cholesterol (A), non-esterified cholesterol (FC) (B), phospholipids (PL) (C), free fatty acids (FFA) (D), triglycerides (TG) (E) were measured enzymatically in apoB depleted serum. HDL associated apolipoproteins ApoA-I (F), apoA-II (G), apoC-II (H), apoC-III (I) and apoE (J) were determined in apoB-depleted serum by immunoturbidimetry.

**Fig 2:**
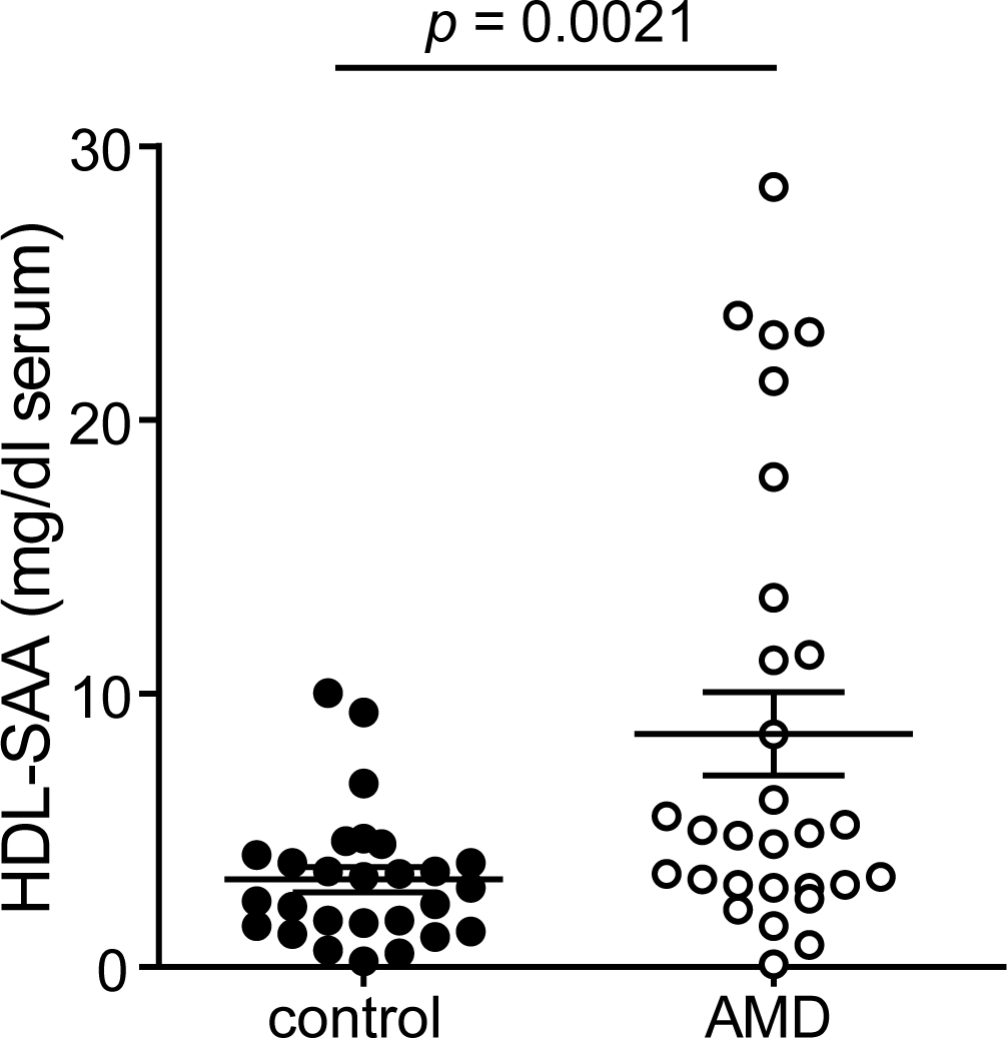
Serum amyloid a levels are increased in AMD patients. Serum amyloid A (SAA) levels in apolipoprotein B (apoB)-depleted sera was quantified by ELISA. Values shown represent means of four independent experiments.
